# Stress testing supply chains and creating viable ecosystems

**DOI:** 10.1007/s12063-021-00194-z

**Published:** 2021-05-29

**Authors:** Dmitry Ivanov, Alexandre Dolgui

**Affiliations:** 1 Berlin School of Economics and Law Supply Chain and Operations Management, 10825 Berlin, Germany; 2 IMT Atlantique, LS2N - CNRS, La Chantrerie, 4 rue Alfred Kastler, 44307 Nantes, France

**Keywords:** Supply chain, Resilience, Viability, COVID-19 pandemic, Stress-test, Ecosystem, Intertwined supply network, Digital supply chain twin, Sustainability

## Abstract

Businesses and governments are becoming increasingly concerned about the resilience of supply chains and calling for their review and stress testing. In this conceptual essay, we theorize a human-centred ecosystem viability perspective that spans the dimensions of resilience and sustainability and can be used as guidance for the conceptualization of supply chain resilience analysis in the presence of long-term crises. Subsequently, we turn to the technological level and present the digital supply chain twin as a contemporary instrument for stress testing supply chain resilience. We provide some implementation guidelines and emphasize that although resilience assessment of individual supply chains is important and critical for firms, viability analysis of intertwined supply networks and ecosystems represents a novel and impactful research perspective. One of the major outcomes of this essay is the conceptualization of a human-centred ecosystem viability perspective on supply chain resilience.

## Introduction

Increased interest in supply chain risks and resilience is usually born out of a crisis. The COVID-19 pandemic has been a long-term supply chain crisis that has revealed a lack of preparedness and insufficient recovery capabilities across numerous industries and sectors. The supply chain and operations research community has responded to the pandemic by creating a profound and strong research record concerning supply chain resilience and management across different pandemic stages. This research covers, for example, the prediction of pandemic impacts (Ivanov 2020a; Singh et al. 2021; Paul and Chowdhury 2021; Queiroz et al. 2020), the reaction of supply chain operations and performance during the pandemic (Choi 2021; Ghadge et al. 2021; Ivanov 2021; Nagurney et al. 2021), and post-pandemic recovery (Ivanov 2021b). At the same time, businesses and governments have recognized the urgent need to review the resilience of supply chains and stress test and enhance their resilience in the future (Simchi-Levi and Simchi-Levi 2020; Barribal et al. 2021).

Both instantaneous disruptions (i.e., those triggered by some single-point-failure interruptions in material flows such as fires or tsunamis) and long-term crises such as pandemics, financial or political crises, and wars existed long before the term ‘supply chain’ was coined, and manufacturing and logistic firms have always tried to find the best ways of managing their operations in the presence of random, epistemic, and deep uncertainties. The COVID-19 pandemic has been the first long-term global supply chain crisis in the last decades (Ghadge et al. 2013; Ivanov and Dolgui 2020; Pavlov et al. 2020; Queiroz et al. 2020). From 1980 to 2020, transformations of production from insourcing to outsourcing, from local to global, and from redundant to lean have been observed, and the paradigm of supply chain management has emerged and rapidly grown. Across different stages of the COVID-19 pandemic, manufacturing and logistics have coped with market, supply, and environmental uncertainties (Choi 2020; Gupta et al. 2020; Ivanov and Das 2020; Aldrighetti et al. 2021; El Baz and Ruel 2021; Sodhi et al. 2021; Yu et al. 2021).

Recent research has posited the need for rethinking supply chain resilience from positions of viability, reconfigurable supply chains, and socio-ecological and open system perspectives – learning from and thinking beyond the COVID-19 pandemic (Dolgui et al. 2020b; Hosseini et al. 2020; Ivanov 2020b; Ivanov and Dolgui 2020b; Azadegan and Dooley 2021; Ruel et al. 2021; Wieland and Durach 2021). In this conceptual essay, we contribute to this debate by theorizing a human-centred ecosystem viability perspective that spans the dimensions of resilience and sustainability and can provide guidance for the conceptualization of supply chain resilience analysis during massive, long-term crises. Subsequently, we turn to the technological level and present the digital supply chain twin as a contemporary instrument for stress testing supply chain resilience. We provide some implementation guidelines and stress that, while resilience assessment of individual supply chains is important and critical for firms, viability analysis of whole ecosystems represents a novel and impactful research perspective for supply chain resilience.

This paper is organized as follows: In Sect. 2, we elaborate on the degrees of order and chaos related to different uncertainty levels. Section 3 presents the notions of viability, ecosystems, and intertwined supply networks and presents our conceptualization of a human-centred ecosystem viability perspective on supply chain resilience. In Sect. 4, we discuss the ripple effect in supply chains during the pandemic. Section 5 elaborates on the design and application of digital supply chain twins for stress testing the resilience of supply chains. Section 6 offers some managerial implications. We conclude with some summary remarks and a discussion of some open research questions and future research directions in Sect. 7.

## Uncertainty, order, and chaos

Disruption is considered a high-impact–low-frequency event (Kaur and Singh 2021; Kinra et al. 2020). The appearance and consequences of disruptions are difficult to anticipate and predict (Altay et al. 2018; Demirel et al. 2018; Dubey et al. 2019, 2021; Pavlov et al. 2019; Ivanov 2020; Sawik 2020; Gupta et al. 2021; Lücker et al. 2021). There are three different types of uncertainty and associated disruptions (Klibi et al. 2010; Ivanov 2021a). ‘Random disruptions’ belong to the category of *known-known* uncertainty – we know that such events can happen, when they can happen, and how likely they are. For example, each summer, countries (and the associated suppliers) in Southeast Asia are hit by typhoons. ‘Hazard disruptions’ are those about which there is *known-unknown* uncertainty – we know that such events can happen but we do not know when they will happen and what their impact will be. An example is the continuously existing danger of earthquakes in Japan, which is known but hardly predictable. Finally, ‘deep disruptions’ typically exhibit *unknown-unknown* uncertainty – we do not know what can happen, when, and what the consequences could be (Paul and Venkateswaran 2020). The deep uncertainty associated with deep disruptions represents the most complex case for decision-making (Table 1).

**Table 1:** Uncertainty characteristics in supply chains

Uncertainty type	Knowledge about uncertainty	Disruption examples	Disruption type	Uncertainty analysis	Specific effects
Random	known-known	Demand fluctuations	Event	Stability	Bullwhip effect
Epistemic / Hazard	known-unknown	Natural disasters	Event	Resilience	Ripple effect
Deep	unknown-unknown	Pandemic	Crisis	Viability	Survivability

Organization, design, and management principles for supply chains are different at different uncertainty levels. The COVID-19 pandemic is one example of a scenario with this deep uncertainty. Of course, pandemics have occurred in the past and public health researchers have warned about the dangers of pandemics for years. However, it does seem true that the COVID-19 pandemic was less predictable than, e.g., earthquakes in Japan in 2011. So, comparing these two cases helps to illustrate the difference between the epistemic and deep types of disruptions/uncertainty.

Random uncertainty (e.g., known-known scenarios such as demand fluctuations) and epistemic uncertainty (e.g., known-unknown scenarios such as natural disasters) have been extensively studied in supply chains (Kamalahmadi and Parast 2017; Niu et al. 2019; Li et al. 2020; Shekarian et al. 2020). For example, bullwhip and ripple effects research has been developed (Dolgui et al. 2020a; Ghadge et al. 2021). But deep uncertainty (e.g., unknown-unknown scenarios such as a pandemic) has received much less attention so far.

**Fig. 1:**
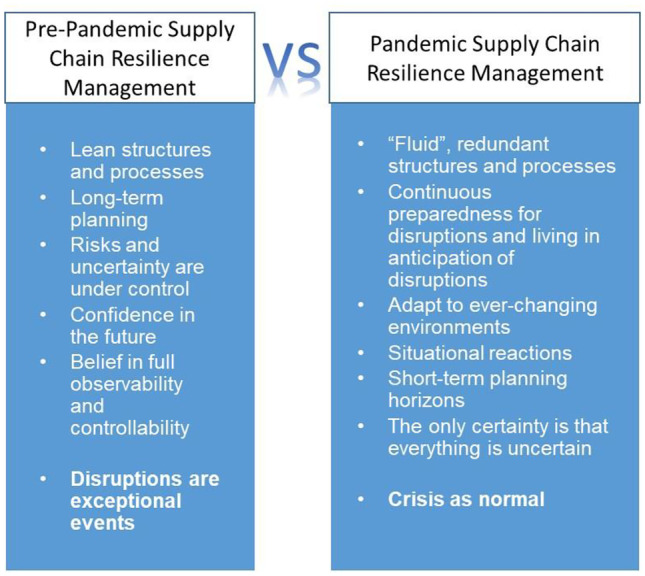
Supply chain resilience management: pre-pandemic vs. pandemic perspectives

Deep uncertainty destabilizes a system and its management and can result in supply chain chaos (Demirel et al. 2019). Different degrees of risk aversion and different understandings of order and chaos, as well as differing adaptability in the speed of thinking and handling, are common when comparing SC management across different uncertainty levels (Fig. 1).

Relative stability in demand and supply in some supply chains over decades led to the formation of a crisis-free management mentality, belief in having risks and uncertainty under control, long-term planning, rigid and lean network structures and planning paradigms – it was all turned upside down during the COVID-19 pandemic. The pandemic challenged supply chain management by introducing a novel and distinct context of order and chaos, controllable and uncontrollable, rigid and fluid, fixed and adaptable, and certain and uncertain (Ivanov 2021b).

During the COVID-19 pandemic, the management mentality has been characterized by a sense of crisis. The need to continuously prepare for disruptions, and living in anticipation of disruptions and continuous change instead of long-term stability, have led to an ability to adapt becoming a central supply chain management perspective. Adaptability and survivability became a normal, rather than an exceptional, state (Ivanov and Dolgui 2020b).

Adaptability as a ‘new normal’, instead of stability and long-term planning, became a deep challenge for firms accustomed to well-organized supply chains, long-term planning, lean structures and processes, and a general confidence in the future and belief in full observability and controllability. Having previously considered disruptions as exceptional events, those supply chains have experienced unprecedented shocks during the long-term pandemic crisis. Because some sentiments exist toward future developments in an increasingly uncertain environment, some research hypothesizes the need for rethinking and reinventing supply chain management to create reconfigurable, viable, and adaptive supply chains and intertwined supply networks (Ivanov 2020b; Ivanov and Dolgui 2020b; Ruel et al. 2021; Wieland 2021; Wieland and Durach 2021). A number of interesting and novel questions arise:

- What is the role of adaptability in the resilience and viability of supply chains, and how can inherent adaptability be implemented while maintaining profitability?
- Should we change from a long-term planning paradigm to a situational reaction paradigm, or can we control the uncontrollable?
- Is planning under chaotic conditions helpful or counterproductive?

Given the scope and scale of future severe threats, they should no longer be considered exceptional events; instead, they should be considered permanent elements of any decision-making environment. Given this, there is a call for the research community to develop strategies, paradigms, and modelling and optimization techniques that account for settings containing deep uncertainty as a ‘normal’ condition. Such research could guide firms in transforming their supply chains and building adaptable, reconfigurable, resilient, and viable value creation systems to reliably provide society and markets with critical services and products on a long-term scale. These transformations require thorough methodological guidelines in order to support decision-making related to long-term supply chain crises involving epistemic and deep uncertainty about current and future developments. To this end, there is also a call for management science research community and further empirical studies to explore and explain supply chain viability phenomena (Ruel et al. 2021).

## Viability, ecosystems, and intertwined supply networks

Resilience is a supply chain’s ability to bounce back once disrupted (Blackhurst et al. 2011; Hosseini et al. 2019; Pettit et al. 2019). Wieland and Durach (2021) note that this principal possibility of returning to an ‘old’ normal state has frequently been taken for granted. The pandemic context is different. In many cases, adaptation to the ‘new normal’ was the only way to survive (Ivanov 2021). This novel context has increased interest in supply chain resilience, and moreover, in the viability of the whole ecosystem of intertwined supply networks (Ivanov and Dolgui 2020b; Ruel et al. 2021).

Triggered by the COVID-19 pandemic, governments plan to review resilience of most critical supply chains such as semiconductors, high-capacity batteries, strategic materials (for example, rare earth elements), and pharmaceuticals, followed by agriculture, commerce, defence, energy, health and human services, homeland security, and transportation (Barribal et al. 2021). Resilience is also one of the central perspectives of the EU’s ‘Recovery plan for Europe’ and the associated NextGenerationEU program.

Identification of critical supply chains is a challenging task. As noted on 23 March 2020 by Vincenzo Boccia, the president of Confindustria in Italy (Agi 2020), it is very difficult to overcome the epidemic crisis and determine the most essential SCs to ensure survivability because ‘suppliers in the automotive sector are at the same time producers of valves for respirators’.

To derive a framework of critical ecosystems, we suggest relying on the Classification of Individual Consumption According to Purpose (COICOP) reference classification developed by the United Nations. The COICOP is composed of 15 categories of human needs which can be aggregated in the following way:

- Food and beverages
- Clothing and footwear
- Housing, water, electricity, gas, and other fuels
- Furnishings, household equipment, and routine household maintenance
- Health
- Transport
- Information and communication
- Recreation, sport, and culture
- Education services

The COVID-19 pandemic impacts have been seen in supply chains associated with each of these categories. Chowdhury et al. (2020) and Singh et al. (2021) identify pandemic impacts on food supply chains. Nagurney (2021) and Sodhi et al. (2021) illustrate the impacts of the pandemic on the healthcare sector and the associated capacity and labour availability challenges for supply chains. Loske (2020) and Choi (2020) uncover transportation impacts of the pandemic.

The example of the COVID-19 pandemic shows that in case of extraordinary events, supply chain resistance to disruptions must be considered at the scale of survivability or viability to avoid supply chain and market collapses and to secure the provision of goods and services (Ruel et al. 2021). According to Ivanov and Dolgui (2020b), ‘viability is a behavior-driven property of a system with structural dynamics. It considers system evolution through disruption-reaction balancing in the open system context. The viability analysis is survival-oriented at a long-term scale’. Ivanov (2020b) defines viability as an ‘ability of a supply chain to maintain itself and survive in a changing environment through a redesign of structures and replanning of performance with long-term impacts’.

The viable supply chain model and its associated frameworks were proposed by Ivanov (2020b) and comprise the supply chain itself; the intertwined supply network (ISN), which is an ‘entirety of interconnected supply chains which, in their integrity secure the provision of society and markets with goods and services’ (Ivanov and Dolgui 2020b); a digital supply chain, which represents a combination of the physical SC; a cyber-physical system and a digital supply chain twin (Cavalcante et al. 2019; Panetto et al. 2019; Ivanov and Dolgui 2020a; Frazzon et al. 2021); and a business ecosystem responsible for securing society’s needs in line with natural, economic, and governance interests.

In this vein, the notion of a viable supply chain integrates the angles of sustainability and resilience and extends them to survivability. They have been seen in the literature as crucial avenues for rethinking and reinventing supply chain management after the pandemic (Brandenburg and Rebs 2015, Dube et al. 2015, Ivanov 2018, Pavlov et al. 2019, Sarkis 2021). The viable supply chain framework can be of value for decision-makers seeking to design supply chain networks, processes, information, and financial systems that can be profitable during positive times, resilient enough to sustain and recover after disruptions, and sustainable during times of long-term, global disruptions with societal and economic shocks.

Viability is concerned with intertwined supply networks ‘that encapsulate entireties of interconnected supply chains, which, in their integrity, secure the provision of society and markets with goods and services’ (Ivanov and Dolgui 2020b). From the position of viability, the ISNs as a whole provide services to society (e.g., food service, mobility service, or communication service) that are required to ensure society’s long-term survival. Analysis of survivability at the level of the ISN requires consideration at the same large scale as analysis of the resilience of individual supply chains. The example of the COVID-19 pandemic clearly shows the necessity of this new perspective.

Ruel et al. (2021) elaborated in detail on commonalities and differences between resilience and viability of supply chains. In particular, they noted that “supply chain viability can be viewed from an overarching adaptation perspective that extends the supply chain resilience notion of a closed-system, “bounce-back” view, with a viable, open supply chain system perspective incorporating "bounce-forward-and-adapt” options”. Moreover, Ivanov (2021a, Chapter 5) provided a structured comparison of supply chain resilience and viability concluding that viability is an extended resilience perspective. A supply chain can be considered viable if it is able to maintain an ecosystem balance (i.e., to achieve homeostasis) at different uncertainty exposure levels. For example, in conditions of epistemic/hazard uncertainty, resilience management is primarily concerned with sustaining and recovering from the disruptions to fulfil the demand. In conditions of deep uncertainty, viability management focusses on survivability, which is intended to secure the provision of products and services to fulfil the minimum needs of the economy and society.

The principal ideas of the viable supply chain and ISN are adaptable structural supply chain designs for situational supply–demand allocations and, most importantly, the establishment and control of adaptive mechanisms for transitions between the structural designs (Ivanov 2021a). The viable supply chain model can help firms guide their decisions about the recovery and rebuilding of their supply chains after global, long-term crises such as the COVID-19 pandemic.

**Fig. 2:**
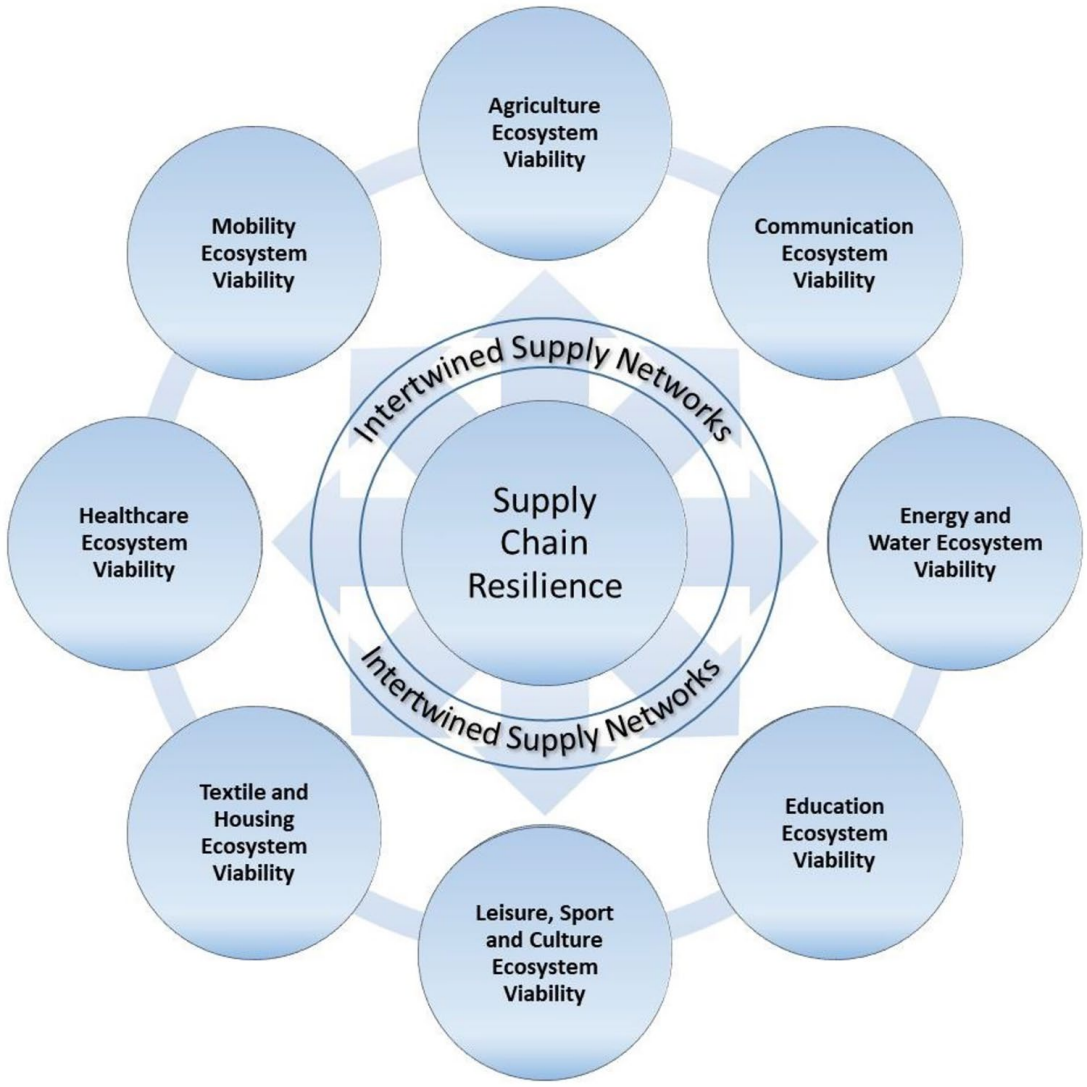
Human-centred ecosystem viability perspective on supply chain resilience

We will now merge the three major topics discussed above – the UN’s classification of major human needs, supply chain viability, and the ecosystem view and synthesize the human-centred ecosystem viability perspective on supply chain resilience (Fig. 2).

Under conditions of long-term uncertainty and lack of resources (i.e., in a crisis), the overall success of the system (i.e., the society) depends on the viability of its critical ecosystems and the concentration of the system’s resources on the development and resilience of its strongest elements. Following the UN’s classification of major human needs, we propose to consider eight major ecosystems, which form a human-centred viability perspective of supply chain resilience:

- Agriculture
- Communication
- Energy and water
- Education
- Leisure, sport, and culture
- Mobility supply chain
- Textile and housing
- Healthcare

To ensure the viability of each ecosystem, society, and economic development, first, the ISNs and associated supply chains in each ecosystem should be identified and analysed. Second, supply chain resilience should be stress tested and enhanced. As such, the following four principles build the agenda of viability:

- Identify major human needs
- Identify ISNs for these needs and the associated ecosystems
- Identify supply chains within ISNs
- Test supply chain resilience

While resilience assessment of individual supply chains is important and critical for firms, analysis of ecosystem viability represents a novel and impactful research perspective. As such, the human-centration perspective can be considered twofold. On one hand, it is an additional ingredient, i.e. putting the needs of humans as one of the central elements of supply chain management practices that will guarantee the viability of the supply chain in the face of a crisis. On the other hand, the human-centration also aims to guarantee the survival of humans when hit by a crisis.

## The ripple effect in supply chains during the COVID-19 pandemic

One specific stressor of supply chains during the pandemic has been the ripple effect – the disruption propagation through the network (Ivanov et al. 2014; Dolgui et al. 2018; Li et al. 2021; Llaguno et al. 2021).

**Fig. 3:**
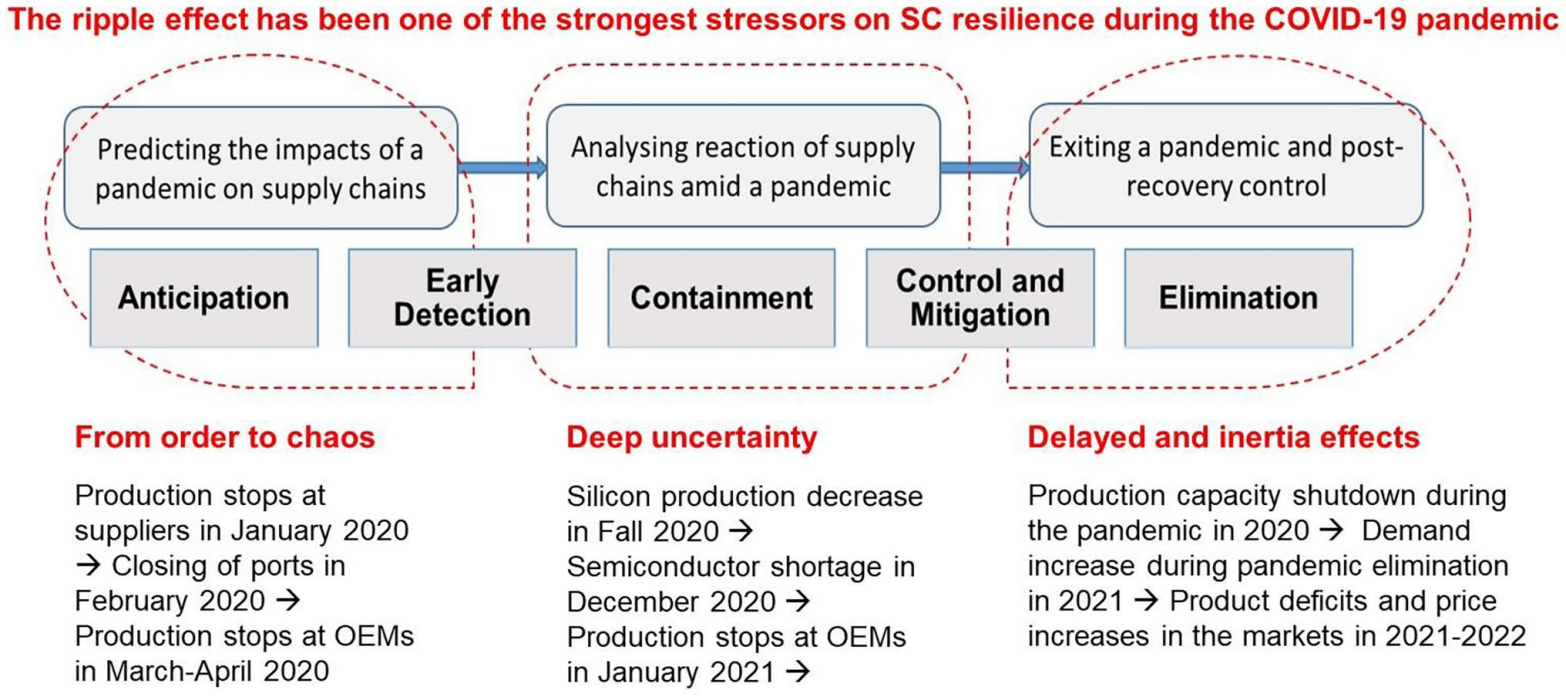
Ripple effects during the COVID-19 pandemic (based on Ivanov 2021b and Ivanov and Dolgui 2021)

Ripple effects have occurred with greater frequency at different pandemic stages (Fig. 3). Haren and Simchi-Levi (2020) point to the ripple effects during the COVID-19 pandemic. The ripple effect then grew substantially, adversely affecting almost all industries and services worldwide (Singh et al. 2021; Ivanov 2021a, 2021; Ruel et al. 2021). Shead et al. (2021) provide evidence of multiple ripple effects that occurred in semiconductor supply chains during the pandemic. Ivanov (2021b) draws attention to some delayed effects and aftershock risks in supply chains that ‘can result in highly destabilized production–inventory dynamics and decreased performance in the post-disruption period causing product deficits in the markets and high inventory costs in the supply chains’.

## Stress testing supply chain resilience using digital twins

With the help of anyLogistix supply chain simulation and optimization software, a digital SC can be designed. Figure 4 shows the structure of a digital SC twin created for disruption analysis using anyLogistix (Ivanov and Dolgui 2020a).

**Figure:**
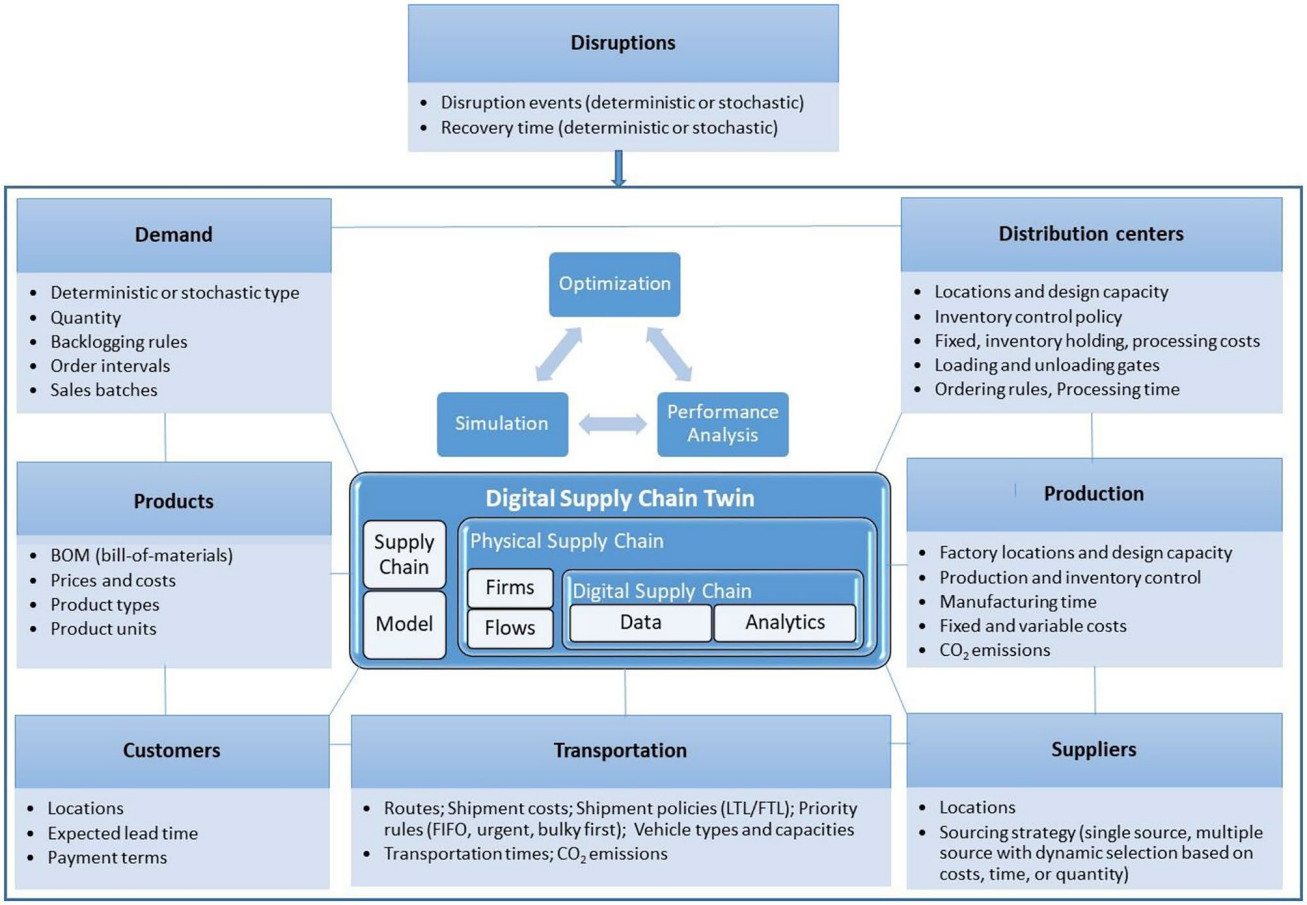
**Fig. 4 **Digital supply chain design for disruption analysis using anyLogistix

anyLogistix is a supply chain simulation and optimization software. It is used for green- and brownfield analysis in facility location planning, network optimization with associated location-allocation planning of material flows, and simulation of supply chain dynamics based on some inventory, production, sourcing and transportation control policies. anyLogistix allows to represent the whole supply chain network with customers, factories, warehouses and suppliers and associated data about locations, demand, capacities, inventory, flows, orders, lead times, CO2 emissions, etc.

The digital SC twin built in the anyLogistix encompasses three major perspectives – the network, the flows, and the parameters. The supply chain network can be designed using different location objects, such as customers, distribution centres (DCs), factories, and suppliers. The flows in the network can be flexibly arranged to represent the specifics of different supply chains. The flows are associated with some design (i.e., maximum) capacities in production, warehouses, and transportation and controlled by associated production, inventory, sourcing, and shipment policies. These policies can be flexibly adapted to the specifics of the SC and its management rules. Finally, different operational parameters, such as demand, lead time, and control policies’ thresholds (e.g., reorder point, target inventory, and minimum vehicle load), can be defined. With that functionality, a digital model of a physical SC (i.e., a digital SC) can be created and used for optimization and simulations to analyse SC operations and performance dynamics under disruptions.

Simulation models for stress testing of supply chain resilience developed in the anyLogistix digital twin have been presented in previous literature (Ivanov 2017; Dolgui et al. 2020a; Ivanov and Rozhkov 2020; Singh et al. 2021). The resilience stress test models are based on observation of disruption impacts on supply chains, understanding the reasons for operational and performance disruptions, and testing different strategies to enhance resilience. The models comprise five control loops: demand, lead time, continuous inventory control with a reorder point and a target stock setting, production control, and transportation control.

Consider an example in line with Ivanov and Dolgui (2020b): Fig. 5 illustrates three major areas of supply chain disruption risk management that are covered in the proposed digital twin – disruption identification, disruption modelling, and disruption impact assessment.

**Figure:**
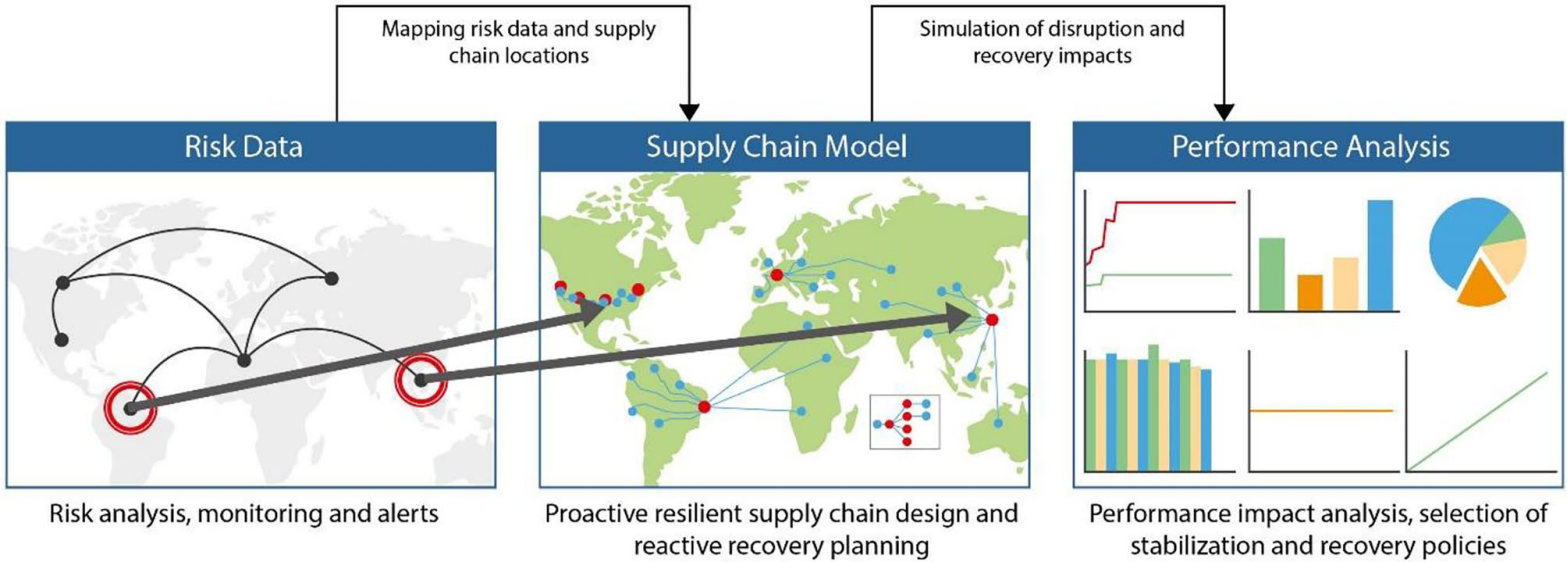
**Fig. 5 **Interrelations between risk data, modelling, and performance analysis (based on Ivanov and Dolgui 2020a)

Figure 5 shows the mapping of risk scenarios, the supply chain design and flows, and the performance impact analysis. During the stress testing process, disruption scenarios are first built (Pavlov et al. 2019) and are then used for supply chain resilience analysis in simulation and optimization models. The digital twin-based resilience analytics system is used to search for bottlenecks that might affect supply chain resilience. The simulation can subsequently be run to observe the impact of disruptions on supply chain performance. Moreover, some recovery policies, such as the activation of alternative supply chain designs during the disruption can be simulated.

In case of using external databases, disruption events and supply chain parameters can be updated automatically every time when the modelling process starts. As such, the real-time digital supply chain twin can be created enhanced by and contributing to end-to-end supply chain visibility (Dubey et al. 2020; Zouari et al. 2021). Finally, resilience analytics can be used as a data-driven learning system to use past experiences to manage future disruptions, thus utilizing cyber-physical, artificial intelligence, and machine learning principles and technologies (Gunasekaran et al. 2016, Cavalcantea et al. 2019, Ivanov et al. 2019, Panetto et al. 2019, Brintrup et al. 2020, Currie et al. 2020, Lohmer et al. 2020).

## Managerial implications

From the practical management point of view, several important questions such as complexity and performance measures for stress testing supply chains and mechanisms to create viable ecosystems need to be clarified. We elaborate on these issues in this section. One major issue in stress testing the whole supply chain network is its complexity. Stress testing of the multi-tier, large-scale network requires high degree of visibility into the network structure. Besides, such an analysis is usually done at quite an aggregate level considering only network structures without parametrized flows and production-inventory control policies. The attempts to detail production-inventory control policies frequently lead to the situation when we can stress test only some fragments of real networks. As such, a balance between network complexity and granularity of the operational level need to be considered (Zhao et al. 2019; Li et al. 2021). The importance of this balancing grows when we go beyond supply chain resilience and focus on the ecosystem viability.

Another important aspect in stress testing supply networks and ecosystem viability is performance measurement. While supply chain resilience measures have been established, there is an acute need to create a performance management system related to viability of intertwined supply networks and ecosystem viability (Ivanov 2021a; Ruel et al. 2021). Viability assessment is closely related to the mechanisms of designing the intertwined supply networks and business ecosystems. The key role in these areas is played by digital twins and end-to-end visibility in order to facilitate data-driven modeling and analysis.

Finally, the COVID-19 pandemic as the most severe stress to modern value creation systems to date is only one of the macro challenges that supply chain networks are currently coping with – and ignoring at times (Fig. 6).

**Figure:**
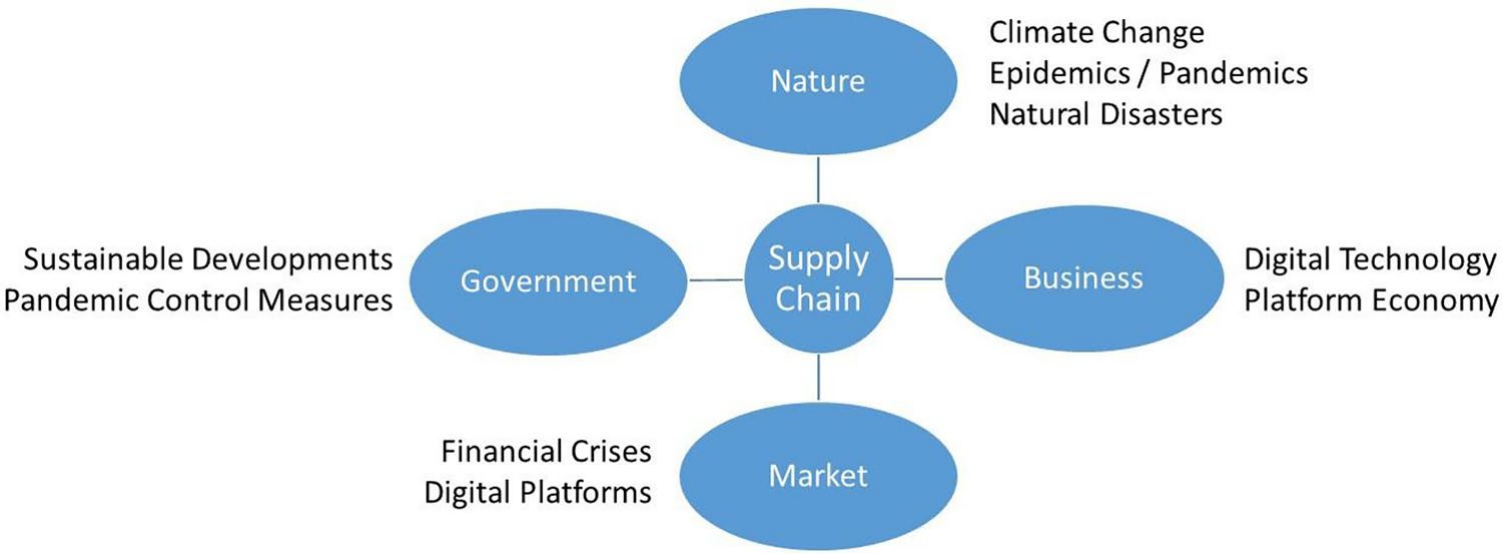
**Fig. 6 **Triggers of supply chain structural dynamics

The simultaneous existence of different disruptive stressors – both of a positive nature (e.g., governmental initiatives supporting sustainable manufacturing, and business-driven digital technology and data analytics developments) and of a negative nature (e.g., nature-related stressors such as natural catastrophes, epidemics/pandemics, and climate change and market disruptions such financial crises) – raises a number of concerns including the following:

- What is the optimal design of a supply chain network – the most efficient, the most resilient, the most sustainable, or the most adaptable one?
- Is it possible to adapt lean and static supply chains?
- How can supply chains be designed to be both adaptable and efficient?
- How can we design and manage global supply chain networks given the situational availability of some regions for production/logistics activities because of quarantines or climate change-driven severe and long natural disasters?
- How can digital technology and data analytics be utilized to enhance supply chain resilience and viability?
- What is the role of adaptability in the resilience and viability of value creation systems, and how can inherent adaptability be implemented while maintaining profitability?
- Should we change from a long-term planning paradigm to a situational reaction paradigm, or can we control the uncontrollable?
- Is planning under chaotic conditions helpful or counterproductive?
- Just-in-time or just-in-case?
- How can we prepare supply chains to operate under deep uncertainty, and how can we deploy contingency plans in the presence of disruptions and their unpredictable scaling?
- Is ecosystem viability a sum of supply chain resiliencies, and what is the role of intertwined network effects?

These and other managerial implications can be encountered during the transition to the viability management paradigm and require both practical and theoretical consideration.

## Concluding remarks, open research questions, and future opportunities

In this conceptual essay, we have theorized a human-centred ecosystem viability perspective building on the resilience of individual supply chains and extending it through intertwined supply networks toward viable ecosystems. The conceptualization we have provided can be used to understand the needs and develop measures to enhance supply chain resilience in the context of not only single-point-failure events but also massive, long-term crises. On the technological implementation level, we discussed how digital supply chain twins can be used for stress testing supply chain resilience. In sum, one of the major outcomes of this essay is the conceptualization of a human-centred ecosystem viability perspective on supply chain resilience.

We have emphasized that, while resilience assessment of individual supply chains is important and critical for firms, viability analysis of ecosystems represents a novel and impactful research perspective for supply chain resilience. This raises a set of new and open questions for future research in light of the stress testing of supply chain resilience and the viability of ecosystems induced by novel decision-making settings in supply chains in the wake of the COVID-19 pandemic. The context and scope of these settings differ across industries and services but share a set of common attributes such as a crisis-like environment with deep uncertainty about the short- and long-term future and resource shortages and adaptability as a ‘new normal’ in place of stability and long-term planning. The creation of not only efficient and resilient but also *viable* value creation systems capable of production continuity and meeting the fundamental needs of society in the presence of long-term crises is imperative.

With regard to technology, the use of digital supply chain twins and the proactive application of resilience analytics to model supply chain reactions to disruptions and associated recovery policies will increase the probability of society’s survival through long-term crises like the COVID-19 pandemic.

Given the scope and scale of severe threats, we should not consider them exceptional events anymore but instead should see them as permanent elements of any decision-making environment. As a result, there is a call for the research community to develop underlying theories, management principles, and modelling and optimization techniques to account for such settings and guide firms in building adaptable, reconfigurable, resilient, and viable value creation systems and human-centred ecosystems. These and other related questions entail a number of interesting and novel contexts.

To summarize, throughout the COVID-19 pandemic, supply chain resilience and viability have been driven to the forefront of research and practical analysis, entailing numerous novel decision-making settings that go beyond state-of-the-art research related to single-point-failure, instantaneous disruptions. Thus, there is an acute need to develop a new body of research – specifically, resilience analytics that could support decision-making related to long-term supply chain crises in the presence of deep and epistemic uncertainty about current and future developments.

From these research domains, we can expect novel and innovative contributions with high practical relevance that are induced by an industrial and healthcare context. The fields of modelling and optimization under deep uncertainty, network theory, game theory, complex adaptive systems, control theory, digital technology, and data-driven analytics have much to contribute to supply chain resilience development. Organizational theory could be applied to explaining the theorized human-centred ecosystem viability. The next step is innovative and high-quality research related to the modelling and optimization of value creation systems in anticipation of and during long-term crises characterized by inherent epistemic and deep uncertainty. Most centrally, the studies that explicitly incorporate the specifics of epistemic and deep uncertainty – and go beyond resilience to singular-event disruptions of some known probability and random uncertainties – will shape the next few years of supply chain resilience research.
